# Hypertension in the Pediatric Kidney Transplant Recipient

**DOI:** 10.3389/fped.2017.00086

**Published:** 2017-05-01

**Authors:** Olga Charnaya, Asha Moudgil

**Affiliations:** ^1^Division of Pediatric Nephrology, Children’s National Health System, Washington, DC, USA

**Keywords:** kidney transplant, hypertension, pediatric, ambulatory blood pressure monitoring, carotid intima-media thickness, pulse wave velocity, antihypertensive drugs

## Abstract

Hypertension after kidney transplant is a frequent occurrence in pediatric patients. It is a risk factor for graft loss and contributes to the significant burden of cardiovascular disease (CVD) in this population. The etiology of posttransplant hypertension is multifactorial including donor factors, recipient factors, medications, and lifestyle factors similar to those prevalent in the general population. Ambulatory blood pressure monitoring has emerged as the most reliable method for measuring hypertension in pediatric transplant recipients, and many consider it to be essential in the care of these patients. Recent technological advances including measurement of carotid intima-media thickness, pulse wave velocity, and myocardial strain using specked echocardiography and cardiac magnetic resonance imaging have improved our ability to assess CVD burden. Since hypertension remains underrecognized and inadequately treated, an early diagnosis and an appropriate control should be the focus of therapy to help improve patient and graft survival.

## Introduction

Hypertension after renal transplantation is a common phenomenon with an estimated prevalence of 70–90% in adults and 58–89% in children (1–3). Long-standing hypertension has been associated with allograft dysfunction, premature atherosclerosis, and cardiomyopathy (4, 5).

Cardiovascular disease (CVD) is much more common among children with chronic kidney disease (CKD) and end-stage renal disease (ESRD) compared to the general population. CVD accounts for a large proportion of morbidity and mortality in this population (6). Renal transplantation improves CVD risk. Compared to patients awaiting transplantation, transplant recipients experience a substantial reduction in the CVD-associated death rate, especially from adolescence onward while they maintain good graft function (7–11). However, hypertension remains a significant and modifiable risk factor for CVD in pediatric transplant recipients. Studies have shown that transplant patients have better systolic and diastolic function than those receiving hemodialysis or peritoneal dialysis (PD), despite having increased prevalence of hypertension and left ventricular hypertrophy (LVH) (12). In addition to offering transplantation to ameliorate the cardiovascular effects of ESRD, aggressively treating blood pressure in transplant recipients would further reduce the prevalence of CVD.

**Figure 1:**
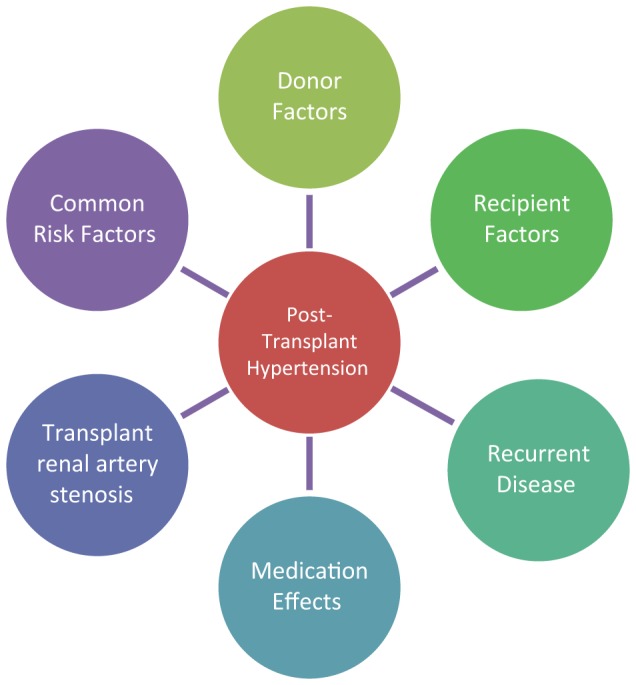
**Etiology of posttransplant hypertension**.

The etiology of posttransplant hypertension is multifactorial encompassing donor-associated factors, side effects of immunosuppressive medications, underlying recipient disease, and possibly genetic factors, as seen in Figure 1. The treatment strategies vary by timing after transplant as well as the pathophysiological contributors to hypertension. This review will cover etiology of posttransplant hypertension, effects of hypertension on the patient, graft survival, assessment methods, and treatment options.

## Etiology of Hypertension

### Donor Factors

It has been well established that several independent donor risk factors predispose the transplant recipient to hypertension including deceased donor, older age, and donor hypertension (13, 14). It is theorized that the increased risk comes from longer cold ischemia time, damaged graft vessels from hypertension, and age-related glomerular dropout in older donors. Due to these reasons, most transplant centers do not accept donors beyond a certain age or those with hypertension for pediatric recipients except in extenuating circumstances. Extended criteria donor kidneys have been associated with increased risk of death from CVD in the adult population and usually are not accepted for pediatric recipients (15).

The kidney donor profile index (KDPI) scoring system, used to evaluate donor grafts, was recently introduced in the United States (16). Donors with lower KDPI have potential for better graft survival, and pediatric recipients get preference for donors with lower KDPI scores, and most pediatric transplant centers will not accept kidneys with high KDPI scores for children. There are no data yet on long-term CVD outcomes for transplant recipients based on donor KDPI scores.

Recently, donor genetic variants have been found to contribute to poor graft function and posttransplant hypertension. Polymorphisms including apolipoprotein L1 (*APOL1*), ATP-Binding Cassette Subfamily B Member 1 (*ABCB1*), Caveolin 1 (*CAV1*), and ATP-Binding Cassette Subfamily C Member 2 (*ABCC2*) have been shown to affect graft survival, hypertension, and calcineurin-induced nephrotoxicity (17–21). Currently, there is no standard for screening for these polymorphisms.

### Recipient Factors

Recipient factors such as recurrence of original disease in the graft and presence of native kidneys are known contributors to posttransplant hypertension. Occasionally, native nephrectomies are performed prior to or at the time of transplant to help with management of hypertension (22). However, this is becoming an uncommon practice because recent data suggest that the benefit is limited (23).

Pediatric patients with pretransplant obesity have significantly higher systolic blood pressure (SBP) and worse glomerular filtration rate (GFR) than children with normal body mass index prior to transplant (24). The incidence of pretransplant obesity is increasing mirroring the rise of obesity in the general pediatric population (25). As in the general population, the development of overweight or obesity after transplantation in children is associated with hypertension and poor glycemic control (26, 27). While these CVD risk factors can more pronounced due to the known side effects of immunosuppressant medications, the effect of obesity on hypertension in the pediatric transplant recipient has been shown to be independent of posttransplant use of steroids (28).

### Effects of Medications

Immunosuppressive medications play a significant role in the development of posttransplant hypertension. It has been well established that corticosteroids induce hypertension by increasing renal salt and water reabsorption and increase in renal vascular resistance. Studies have shown that patients on steroid-minimization protocols and patients undergoing late-withdrawal of steroids showed reduction in hypertension and obesity and improved lipid and carbohydrate metabolism (29).

The incidence of posttransplant hypertension significantly increased after calcineurin inhibitors (CNI) such as cyclosporine and tacrolimus became commercially available for use. A recent Greek cohort of pediatric transplant patients showed that there was an increased risk of hypertension for patients on a CNI-based immunosuppression protocol (14).

The mechanism of CNI-induced hypertension is primarily mediated by glomerular afferent arteriolar constriction leading to increased salt and water retention, in part through upregulation of the thiazide-sensitive Na+-Cl− cotransporter (30, 31). Additional mechanisms include activation of renin angiotensin aldosterone system and secretion of reactive oxygen species (32, 33). An imbalance between vasoconstrictive molecules (endothelin, thromboxane, and prostaglandins) and vasodilatory nitric oxide also contributes to the development of hypertension (34, 35). Finally, prolonged CNI exposure causes increased production of transforming growth factor-β production, which leads to fibrosis and long-term graft damage (36–38). Tacrolimus has become the preferred CNI in clinical practice as it causes less hypertension and hyperlipidemia, as well as improved graft survival when compared to cyclosporine (39, 40).

Previously, it has been shown that mammalian target of rapamycin (mTOR) inhibitors act synergistically with CNI to worsen hypertention (41). Brunkhorst et al. (2015) recently compared the efficacy and safety of an everolimus and low-dose CSA regimen to standard dose cyclosporine and mycophenolate mofetil therapy in 105 pediatric transplant patients. They found that the everolimus group had comparable graft function and survival but more dyslipidemia and arterial hypertension than the control group (42). This suggests that mTOR inhibitors have an effect on posttransplant hypertension independent of or in synergy with the CNI effect, although the precise mechanism is not yet known.

### Transplant Renal Artery Stenosis (TRAS)

Transplant renal artery stenosis is the most common vascular complication after renal transplant, usually presenting 2 months–2 years after transplant (43). Clinical signs are usually worsening or refractory hypertension with or without graft dysfunction (44). It has a reported prevalence of 1–23% and accounts for 1–5% of posttransplant hypertension (45).

Risk factors for TRAS include surgical technique, deceased donor, cytomegalovirus infection, and prolonged ischemia time (46). A recent study investigated the role of genetic polymorphisms in the myosin heavy chain 9 (*MYH9*) gene on TRAS. The product of this gene is heavily expressed in glomeruli, tubular, and renal capillaries as well as arteriolar endothelial cells. Donor organs found to carry the rs3752462 CC variant had a 10.9-fold increase in TRAS compared to the recipients carrying rs5756168 TT variant that had a 3.45-fold decrease in risk (47).

### Graft Dysfunction

#### Recurrent and *De Novo* Glomerular Diseases

Many underlying glomerular diseases in the recipient can recur in the transplant allograft including focal segmental glomerulosclerosis (FSGS), atypical hemolytic uremic syndrome (aHUS), antiglomerular basement membrane disease, systemic lupus erythematosus nephritis, membranous glomerulonephritis (MGN), and membranoproliferative glomerulonephritis. Of these, the most common are aHUS and FSGS, the latter with a 30–50% recurrence risk in the first transplant and 80–100% in subsequent transplants (48). Some transplant patients develop *de novo* glomerular disorders after transplant that may include FSGS and MGN. These glomerular disorders have the potential to cause hypertension, and poor blood pressure control will accelerate renal function decline (49–51).

#### Acute Rejection (AR)

Acute rejection can be T-cell and antibody mediated and is often associated with hypertension. Any transplant patient with sudden onset or worsening of hypertension should be assessed for AR. The hypertension in this case usually responds well to treatment of AR.

#### Chronic Rejection, Also Known As Chronic Transplant Glomerulopathy (TG)

Transplant glomerulopathy causes hypertension through progressive scarring and fibrosis. Recent data showed that non-HLA autoantibodies targeting the angiotensin II type-1 receptor have been linked to hypertension, and treatment with an angiotensin receptor blocker may be beneficial (52–54).

### Common Risk Factors Prevalent in the General Population

**Figure 2:**
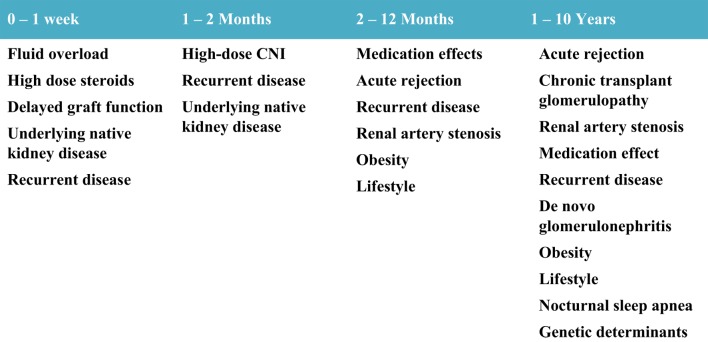
**Etiology of hypertension by time after transplant**.

While patients with ESRD have numerous additional risk factors compared to normal children for developing hypertension, common risk factors for hypertension present in the general population also prevail in kidney transplant recipients. These risk factors include tobacco smoking, illicit drug use, medications for attention deficit hyperactivity disorder, high-salt diet, physical inactivity, obesity, sleep disturbances including obstructive sleep apnea, and genetic determinants of hypertension (55–59). Figure 2 outlines the common etiologies observed for post-transplant hypertension in relation to time after transplant.

## Effect of Hypertension on CVD and Allograft Function

Definitive cardiovascular outcomes such as stroke, myocardial infarction, and death are uncommon in the pediatric age group. Pediatric studies have relied on the use of intermediate endpoints such as LVH as measured by left ventricular mass index (LVMI), carotid intima-media thickness (cIMT), and pulse wave velocity (PWV) for CVD risk stratification. It has been well described that pediatric patients with CKD and ESRD have significantly increased cardiovascular morbidity and mortality compared to their healthy peers (60, 61). CVD accounts for approximately 30% of mortality among pediatric kidney transplant recipients (9).

Long-standing hypertension has been shown to result in LVH and increased LVMI and cIMT, all known risk factors for CVD (2). LVH is prevalent in approximately 50% of patients with CKD and ESRD and in kidney transplant recipients. A single-center study comparing pretransplant echocardiography (ECHO) with posttransplant ECHO showed that interval decrease in indexed SBP was the only predictor of LVMI improvement on multivariate analysis (62). A cross-sectional study in 2004 found that children with a kidney transplant were more likely to have elevated LVMI and diastolic dysfunction than healthy controls (63). However, another study found no difference in systolic or diastolic function between normotensive and hypertensive pediatric transplant recipients using the same tissue Doppler technique (2).

Evidence suggests that hypertension itself, regardless of BP level, is associated with LVH. Specifically, Hamdani et al. showed that treated hypertensive individuals with a normal blood pressure had a greater prevalence of LVH than normotensive individuals not treated with antihypertensive medications. In this same study, a large pediatric cohort of 221 patients evaluated with ambulatory blood pressure monitoring (ABPM), it was noted that patients with normal blood pressure who did not require antihypertensive medications had less allograft dysfunction than patients taking antihypertensive medications and even in those whose BP was in normal range (64). This emphasizes the point that children with pharmacologically controlled hypertension are still at an increased CVD risk compared to normotensive individuals and require close screening for intermediate risk factors and endpoints.

Masked hypertension as determined by ABPM in both patients receiving and not receiving antihypertensive therapy has an estimated prevalence of 25–35% (65). While smaller studies have not been able to find an association between masked hypertension and graft function, a recent study by Hamdani et al. showed that pediatric transplant recipients with masked hypertension had significantly worse graft function than their normotensive peers (66).

A study aimed at assessing CVD risk factors in pediatric transplant patients found that there was a negative correlation between GFR and SBP ± diastolic blood pressure (DBP) on univariate analysis. There was a noted trend of worsening hypertension with increasingly poor graft function on multivariate analysis, but the study was underpowered to detect this effect (67). A Finnish cohort found that only decreased diastolic dipping could predict lower GFR (68). More recently in a Greek cohort, 20-year graft survival was superior for patients without hypertension at 10 years follow-up after kidney transplant compared to those with hypertension (100 vs. 44.4%, *P* < 0.05) (14). As well established in native kidneys with CKD, the presence of poorly controlled hypertension also negatively affects renal graft function over time.

A Dutch cohort study looking at long-term CVD outcomes in kidney transplant recipients (with an average follow-up of 15. 5 years) who developed ESRD as children (0–14 years of age) found that ~50% of males and 40% of females had LVH, 13% had diastolic dysfunction, and aortic valve calcification was seen in 25% males and 12% female patients (69). Duration of PD was independently associated with development of aortic valve calcifications and diastolic dysfunction increased over time and correlated with low GFR. This study also demonstrated an era effect as patients who developed ESRD in the 1970s and 1980s had increased prevalence of CVD prior to more aggressive blood pressure control, ubiquitous use of erythropoietin-stimulating agents, frequent avoidance of aluminum, and limited use of calcium-based phosphorus binders (69). A similar recent Dutch cohort still continued to show era effects in CVD outcomes with improved management of hypertension and hyperlipidemia (70).

In conclusion, children with CKD/ESRD come to transplant with significant burden of hypertension and CVD. With transplantation, their CVD risk improves overall but may be negatively impacted by poor control of blood pressure, by decline in GFR overtime, and by the presence of hyperlipidemia, obesity, and hyperglycemia.

## Blood Pressure Measurement and Assessment of CVD Risk

Screening for hypertension can be achieved by several methods that include casual clinic blood pressure measurement, home and school blood pressure measurement, and 24-h ABPM. ABPM has been shown to be the most reliable method for measuring blood pressure. It also has the added benefit of determining BP load and predicting LVH (71–73). Pediatric patients with ESRD who lack nocturnal dipping as characterized by a decrease of >10% in nighttime blood pressure compared to daytime blood pressure are deemed to be at an increased risk of LVH. However, this was not borne out in another recent study that showed that hypertensive children without ESRD who have nocturnal non-dipping have similar prevalence of LVH compared to hypertensive children without non-dipping (74, 75).

Before widespread use of ABPM, we must recognize its current limitations. Primarily, controversy remains about how to best interpret ABPM results due to lack of diverse normative data. Currently available normative data are based on ABPM measurements of 949 healthy children published by the German Working Group of Pediatric Hypertension (76). All children included in this study were of European descent, relatively few short children (<140 cm) were included, and there was a lack of variability in DBPs within the group raising question about the algorithm used to calculate the normal values.

### Carotid Intimal Media Thickness

High-resolution ultrasonography provides a non-invasive method for measuring cIMT. Increased cIMT, a marker for the development and progression of vascular calcification, has been documented in children as young as 8 years and correlates with duration of CKD, time on dialysis, hyperhomocysteinemia, and increased calcium–phosphorus product (77, 78). A recent meta-analysis showed that pediatric solid organ transplant recipients have increased cIMT compared to healthy controls (79). Increased cIMT has been shown to be a strong risk factor for myocardial infarction and stroke in adults (80). Childhood hypertension has been shown to predict increased adult cIMT by the Childhood Cardiovascular Cohort (i3C) (81).

Hypertension has been shown to have a linear correlation with cIMT in adults (82). Balzano et al. showed that although renal transplant recipients had increased cIMT and LVMI compared to healthy controls, there was no progression or worsening in those who underwent annual ABPM monitoring and had well-controlled hypertension (83). This study is reassuring of the fact that while renal transplant recipients have greater cIMT and LVH than healthy controls, regular blood pressure monitoring and aggressive blood pressure control can halt the progression of these intermediate CVD outcomes.

### Pulse Wave Velocity

Arterial PWV is a sensitive marker of arterial stiffness, which makes it a good surrogate endpoint for CVD (84). Increased arterial stiffness is an independent predictor of survival in the general population and in CKD patients (85). Normative pediatric reference values are available based on a study of 1,000 healthy children, which enabled the calculation of age- and height-specific SD scores (86). This study was performed on patients in Hungary, Italy, and Algeria with ages ranging between 6 and 20 years. Multiple regression analysis showed that age, height, and blood pressure were major predictors of PWV.

Chronic kidney disease patients have been shown to have increased PWV. The recent 4C Study looked at numerous cardiovascular endpoints including PWV and found that 20.1% of CKD patients in their cohort had elevated PWV. In their analysis, the rise in PWV was independent of eGFR and moderately correlated with cIMT (87). Sinha et al. (2015) showed that in children with advanced CKD, only poorly controlled hypertension but not GFR was associated with a change in arterial stiffness when compared to healthy matched controls (88). In addition, the Young Finn study showed that childhood hypertension was related to higher adult PWV (89). In pediatric kidney transplant recipients, PWV has been shown to be increased compared to control patients matched for age and weight/height (26, 90). Since pediatric kidney transplant recipients have ongoing CKD, strict BP control may help to lower PWV and future cardiovascular risk in this population.

### Myocardial Strain Analysis

The majority of coronary artery disease in CKD patients is asymptomatic and may initially present with arrhythmia and sudden death. Assessing the degree of myocardial strain using ECHO or magnetic resonance (MR) imaging can detect more subtle changes of impaired cardiovascular function, thus offering an opportunity for early intervention.

Echocardiographic strain imaging helps to objectively quantify myocardial function (91). An extension of this is global longitudinal strain (GLS) analysis, which can be used to detect subtle changes in left ventricular function. GLS has been shown to be independently associated with all-cause and CV mortality in adult patients with CKD and after renal transplant. It is a more reliable marker than ejection fraction for mortality (92, 93). Pirat et al. demonstrated that ESRD patients with a normal ejection fraction had signs of subclinical myocardial disease as determined by impaired longitudinal, circumferential, and radial strain as well as strain rate (94). In that same study, patients who underwent renal transplantation had improvement in all of these parameters compared to the patients receiving chronic dialysis. The clinical characteristics that negatively affect GLS and basal longitudinal systolic strain were shown to include increased interventricular septal thickness, diabetes, low ejection fraction, increased DBP, and regular dialysis (95). This again highlights the role of hypertension as a modifiable risk factor.

Cardiac MR is a relatively newer imaging modality that can provide additional information about CV risks in the CKD population. Blood oxygen level-dependent cardiac MR can be used to assess myocardial tissue oxygenation. A recent study employing this technique found that patients with CKD and renal transplant without known coronary artery disease had impaired myocardial response to stress, independent of the presence of diabetes mellitus, LVH, and myocardial scar (96). An additional use for cardiac MR imaging is to estimate LVM, which has been shown to closely correlate with autopsy findings of heart size. A recent study compared ECHO and cardiac MR in their ability to measure LVM in hypertensive pediatric patients and found that ECHO generally overestimated the presence of LVM and cardiac MR was a much more reliable method (97).

As these technologies become more readily available and cost-effective in clinical practice, they will be able to offer more reliable tools to assess cardiovascular dysfunction in pediatric transplant recipients.

## Management of Hypertension

### Acute Postoperative Management

There are no published guidelines or recommendations for the immediate postoperative management of kidney transplant recipients. We will describe our center’s practice based on our clinical experience in this section. In the immediate postoperative period (first week), the most likely etiology of hypertension is fluid overload, side effect of high-dose steroid therapy for induction, and native kidney disease. In the first few postoperative days, permissive hypertension is tolerated to ensure adequate graft perfusion especially in small children. Size discrepancy of the donor and recipient arteries and relatively lower blood pressure in the child can compromise early graft perfusion and predisposition to thrombus formation. This necessitates higher patient blood pressures and may require vasopressor support to achieve this.

After a few days postoperatively, excess fluid begins to mobilize and move into the intravascular space, contributing to hypertension. Allowing gradual diuresis back to the patient’s dry weight will often result in decrease of the systemic blood pressure. However, patients frequently need to be restarted or initiated on antihypertensive medications during the postoperative period.

### Long-term Management

A holistic approach to the long-term management of hypertension should be pursued in the pediatric transplant recipient. As the goal of hypertensive management is ultimately to prolong patient and graft survival and decrease CVD, this approach should include close monitoring and management of graft function as well as adherence to medications, diet, and exercise. Many review papers have been published on the pharmacologic management of hypertension (1, 98, 99). The current KDIGO position is that no class of antihypertensive medications is contraindicated in transplant recipients (100). In clinical practice, calcium channel blockers (CCBs) and ACE-I are the most common first-line medications (101–103). While permissive hypertension is tolerated immediately after transplant surgery, the ultimate goal of therapy should be to target normal blood pressures (<90th percentile) and try to achieve control near the 50th percentile for age.

As with hypertension in patients without renal transplant, pharmacologic therapy should be aimed at addressing the underlying etiology. In patients on CNI-based immunosuppression, afferent arteriole vasoconstriction contributes to hypertension, and therefore, a use of CCBs is usually the first-line therapy due to its vasodilatory properties. CCBs have a very good safety profile and have been shown to be efficacious in this patient population for both blood pressure control and improvement of GFR compared to placebo treated patients (101). In non-transplant patients, the benefit of ACE-I in the pediatric CKD population with proteinuria has been well described (51). Less concrete evidence exists for the use of ACE-I in kidney transplant recipients. Knoll et al. performed a multicenter double-blind placebo-controlled study comparing ramipril to placebo and found no benefit in allograft survival in the treatment group. His study was underpowered to detect a difference and may account for their null results (104). A recent meta-analysis to look at the effects of ACE-I on graft survival also found no benefit (105). A small case series in a pediatric population found that ACE-I appeared to stabilize creatinine in patients with chronic allograft dysfunction, but statistical analysis could not support the renoprotective effect of ACE-I on long-term graft survival (106). Meta-analysis by Cross et al. showed that therapy with ACE-I can reduce proteinuria, however there are not enough data to support that this improves graft survival (101). Diuretics are not usually employed in the pediatric transplant population due to the need to maintain adequate hydration; however, a thiazide diuretic may be effective in helping control CNI-mediated hypertension due to its action through the thiazide-sensitive Na-Cl channel (30, 107).

### Dietary Counseling

While pharmacological interventions have an important role in the management of hypertension, dietary and exercise counseling need to be at the forefront of long-term management of transplant recipients.

Diets high in sodium and low in potassium have been shown to aggravate hypertension. In a Belgian study of adult patients, dietary history and urinary sodium and potassium excretion were evaluated. While sodium intake did not differ between the two groups (~10 g/day), patients with controlled blood pressure consumed higher amounts of potassium by regularly eating more fruits and vegetables, which lowered their observed Na+/K+ ratio (108). Asai et al. demonstrated that repeated dietary counseling resulted in a statistically significant reduction in mean 24-h urinary sodium excretion and SBP (109). Similarly, de Vries et al. also showed that dietary sodium restriction in adult kidney transplant recipients resulted in statistically significant reductions in SBP and DBP (110). Both of these studies had small sample size, and therefore, it is impressive that the noted changes in blood pressure were able to reach statistical significance.

In accordance with these findings, published nutritional guidelines for renal transplant recipients include low-sodium diet and weight loss in obese individuals to optimize blood pressure management (111). Counseling of patients should be done routinely, as infrequent counseling has been shown to be not effective (112).

### Systems-Based Approach

Changes and improvement in management of hypertension in the pediatric kidney transplant population can be approached using quality improvement methodology. Because this is a complex multifaceted problem that affects patients’ long-term outcomes and requires multidisciplinary cooperation, this can be addressed by employing the Chronic Care Model as suggested by Hooper and Mitsnefes (113). Recommendations include implementation of clinic visit checklists to ensure that blood pressure, diet, and lifestyle risk factors are addressed regularly at every clinic visit, augmenting the electronic health record (EHR) to calculate blood pressure percentiles, pop-up reminders for annual ABPM screening, and employing novel technology to encourage activity and participation among transplant recipients.

## Conclusion

**Figure 3:**
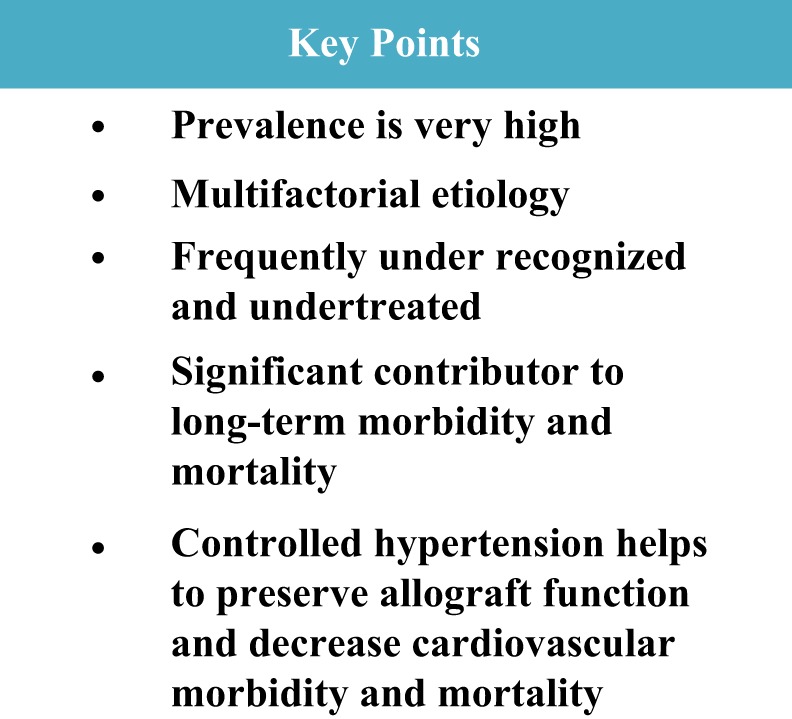
**Key points**.

Hypertension in the pediatric kidney transplant recipients is highly prevalent and often underrecognized and undertreated. This contributes to worse allograft function and long-term risk of CVD in early adulthood and partly accounts for the significantly increased mortality in this patient population. Hypertension is a modifiable risk factor that should be aggressively monitored and treated, as summarized in Figure 3. Annual 24-h ABPM and echocardiography are useful tools to asses CVD risk and monitor blood pressure control. Newer technologies such as measurement of cIMT, PWV, and myocardial strain can be considered to enhance CVD risk stratification. Treatment should include a combination of pharmacological and lifestyle interventions to optimize care utilizing a multidisciplinary team. Currently, quality improvement methodology and EHR technology provide an opportunity to customize workflows and build in templates/reminders to optimize patient care and improve patient outcomes.

## Future Research

There are many gaps in our knowledge in this field that would benefit from further research including but not limited to CVD outcomes based on the donor KDPI; genetic polymorphism of donors affecting posttransplant hypertension in transplant recipients; ABPM normative data of diverse ethnic populations; blood pressure percentile treatment goals for transplant recipients; effect of ACE-I on long-term graft survival, and finally, addressing barriers to adherence at both provider and patient level with adequate treatment plan.

## Author Contributions

OC wrote the manuscript under guidance and supervision by AM. Both parties were directly involved and were the only contributors to the production of the manuscript.

## Conflict of Interest Statement

The authors declare that the research was conducted in the absence of any commercial or financial relationships that could be construed as a potential conflict of interest.
